# Association of image-defined risk factors with clinical features in thoracic neuroblastoma and the development of a prognostic prediction model

**DOI:** 10.3389/fped.2026.1793866

**Published:** 2026-04-22

**Authors:** Chong Shen, Zihao Bai, Kaiqian Zhou, Yujun Ma, Ming Yang, Jirong Qi, Bo Qian, Kaihong Wu

**Affiliations:** 1 Department of Cardiothoracic Surgery, Children’s Hospital of Nanjing Medical University, Nanjing, China; 2 Nanjing Children’s Hospital, Clinical Teaching Hospital of Medical School, Nanjing University, Nanjing, China; 3 Department of Nephrology, Children’s Hospital of Nanjing Medical University, Nanjing, China; 4 Department of Radiology, Children’s Hospital of Nanjing Medical University, Nanjing, China

**Keywords:** IDRFs, image-defined risk factors, neuroblastoma, prediction model, thoracic

## Abstract

**Background:**

Image-defined risk factors (IDRFs) were critical in managing and predicting outcomes for neuroblastoma. This study systematically evaluated baseline clinical features, IDRFs prevalence, and prognosis in thoracic neuroblastoma, explored the association between IDRFs and these features, and developed a nomogram to predict event-free survival (EFS).

**Methods:**

Clinical and prognostic data were collected retrospectively from pediatric patients diagnosed with thoracic neuroblastoma at Children's Hospital of Nanjing Medical University. Logistic regression was used to identify factors associated with IDRFs presence. Kaplan–Meier analysis assessed the effect of IDRFs on survival. Cox regression identified independent predictors of EFS, with model selection based on the Akaike Information Criterion (AIC). A nomogram was developed from these predictors and evaluated using calibration curves, time-dependent AUC curves, and decision curve analysis (DCA).

**Results:**

Total of 105 patients were included, 56 males, with a median diagnosis age of 55 (29, 77) months. The most common diagnosis was ganglioneuroblastoma intermixed (GNBi) (*n* = 49). Preoperative imaging found 93 IDRFs in 64 patients (60.95%), with infiltrative IDRFs being the most prevalent. 65.63% of patients had only one IDRF. Logistic regression showed that total protein (TP) ≥ 69.90 g/L and maximum tumor diameter (MTD) ≥ 5.50 cm independently predicted IDRFs presence. Among 85 patients with non-ganglioneuroma diagnoses, IDRFs did not significantly affect overall survival (OS) (*p* = 0.289), but were linked to worse EFS (*p* < 0.05). Cox regression identified infiltrative IDRFs, vascular IDRFs, LDH ≥273.00 U/L, and TP ≥ 70.00 g/L as independent risk factors for poor EFS. Nomogram based on these variables showed favorable discrimination with C-index = 0.77, good calibration, and clinical utility.

**Conclusions:**

Thoracic neuroblastoma had unique demographic and clinical features. TP levels and tumor size were associated with the presence of IDRFs, which significantly affected EFS. The nomogram accurately predicted EFS and held potential for clinical utility.

## 1 Introduction

Neuroblastoma is one of the most common extracranial malignant tumors in pediatric populations, predominantly affecting children under 5 years old. It accounted for approximately 8%–10% of all childhood malignancies (1). The disease arises from primordial neural crest cells, with the most frequently affected anatomical sites being the adrenal medulla, retroperitoneum, mediastinum, and cervical region (2, 3). Neuroblastoma exhibits significant heterogeneity in clinical outcomes. While a minority of cases may experience spontaneous tumor regression, the majority of pediatric patients faced a poor prognosis. Consequently, neuroblastoma contributed to approximately 12% of cancer-related deaths among children under 15 years old (4–6).

The aggressive biological behavior of neuroblastoma can increase the surgical risk and therapeutic challenges. To address this, the International Neuroblastoma Risk Group (INRG) introduced the International Neuroblastoma Risk Group Staging System (INRGSS) in 2009, which was based on image-defined risk factors (IDRFs) (7). This staging system did not depend on surgical findings and enabled preliminary staging of patients before pre-treatment, thereby facilitating early implementation of individualized therapeutic strategies.

However, the INRGSS classification system was limited to classifying tumors into stages L1 and L2 based on the presence or absence of IDRFs. There remained a lack of consensus regarding the prognostic significance of different types and quantities of IDRFs. Given the considerable variation in the incidence of neuroblastoma across anatomical locations, most clinical investigations had concentrated on adrenal and abdominal tumors, with limited systematic studies focusing on thoracic neuroblastoma. Existing evidence suggested that thoracic neuroblastoma exhibited distinct clinical and tumor biological characteristics compared to abdominal neuroblastoma, as well as differences in the characteristics and incidence of IDRFs (8, 9). This study aimed to retrospectively analyze clinical data from a single institution to characterize the clinical features, IDRFs distribution, and prognostic outcomes of pediatric patients with thoracic neuroblastoma. Furthermore, we evaluated the association between IDRFs and clinical features, and assessed their potential prognostic value, with the objective of providing clinical evidence to support the validation and potential refinement of the INRGSS staging system.

## 2 Materials and methods

### 2.1 Study population

This study retrospectively collected data from patients with thoracic neuroblastoma treated at the Children's Hospital of Nanjing Medical University between January 2017 and June 2024. Before the operation, a comprehensive evaluation of the tumor's relationship with surrounding tissues and organs should be conducted based on contrast-enhanced CT and/or MRI, as well as other imaging modalities. For those who could not undergo single-stage tumor resection, neoadjuvant chemotherapy was given, and the postponed or second-stage surgery was determined based on the extent of tumor regression. The inclusion criteria for this study were as follows: (1) age < 18 years, with no sex restrictions; (2) the primary tumor was located in the thoracic region; (3) postoperative pathology confirmed the diagnosis of neuroblastoma; (4) complete clinical information, imaging data, and follow-up records. This study has been approved by the Hospital Ethics Committee of the Children's Hospital of Nanjing Medical University (Permission No. 202205047-1).

**Figure 1:**
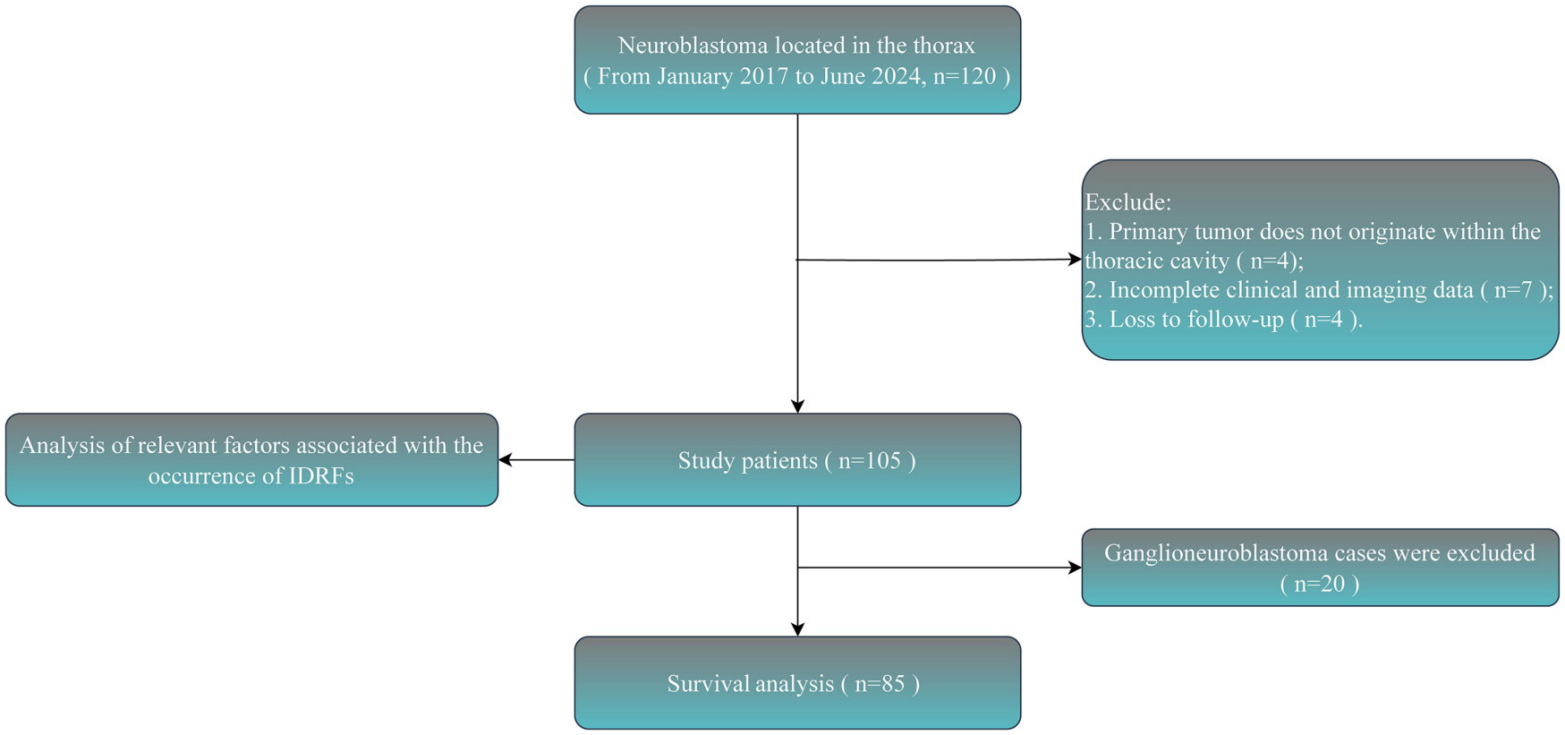
Flowchart for the selection procedure of patients with thoracic neuroblastoma.

### 2.2 Data collection

The sample size for this study was determined based on the availability of data within our dataset. The collected clinical variables included the sex, age at initial diagnosis, and the first available preoperative serum levels of neuron-specific enolase (NSE), lactate dehydrogenase (LDH), total protein (TP), high-density lipoprotein (HDL), and hemoglobin concentration (Hb). Imaging data encompassed preoperative and postoperative CT and MRI scans. These imaging data were independently reviewed by two trained professionals to assess IDRFs. Discrepancies or indeterminate imaging features were resolved through consensus evaluation by experienced senior radiologists. The maximum tumor diameter (MTD), defined as the longest dimension of the tumor in its largest cross-sectional image, was recorded as the primary tumor size. Pathological classification was performed in accordance with the International Neuroblastoma Pathology Classification (INPC) system (12), based on postoperative histopathological findings. Tumor staging was conducted using the International Neuroblastoma Staging System (INSS) to determine the postoperative clinical stage.

In 2009, the International Neuroblastoma Risk Group (INRG) described more than twenty types of IDRFs occurring in various anatomical regions of the human body. Among these, Delforge et al. summarized the IDRF of thoracic neuroblastoma and classified them into four major categories: infiltrative, vascular, neurological, and extensive (13).

Overall survival (OS) was defined as the interval from the date of diagnosis to either the date of death or the last follow-up. Event-free survival (EFS) was defined as the time from diagnosis to the occurrence of the first adverse event, including tumor progression, recurrence, secondary malignancies, or death from any cause, with censored observations determined by the date of the last follow-up. Patients who were lost to follow-up were classified based on their last documented clinical status, with those still alive at the time of the last follow-up considered as censored observations. The follow-up endpoint for this study was set at December 2024.

### 2.3 Statistical analysis

Data were processed, analyzed, and visualized using SPSS (version 26.0) and R software (version 4.3.3). Continuous variables were assessed using independent-sample *t*-test or Mann–Whitney *U* test, while categorical variables were evaluated using the Chi-square (*χ*^2^) test or Fisher exact test. *p* < 0.05 was considered statistically significant. The cut-off values of the laboratory variables and the maximum tumor diameter determined in this study were achieved by using the R package “Cutoff” (version 1.3). Univariate logistic regression analysis was initially conducted to identify potential factors associated with IDRFs. Variables with *p* < 0.10 were subsequently incorporated into a bidirectional stepwise multivariate logistic regression model to identify independent predictors. Survival and prognostic data were analyzed using the Kaplan–Meier method and Log-rank test. Univariate and multivariate survival analyses were performed using the Cox proportional hazards model. Independent risk factors influencing event-free survival (EFS) were selected based on the minimum Akaike Information Criterion (AIC) and included in the development of nomogram model. Given the extremely low proportion of N-MYC amplification in this study cohort (*n* = 2), this well-established prognostic biomarker was excluded from the multivariable prognostic analysis to avoid model instability and overfitting.

Nomogram, also referred to as a nomograph, is a widely used graphical tool that enables the estimation of individual probabilities of disease occurrence or prognosis (14, 15). The discriminative ability of the nomogram was evaluated using the concordance index (C-index) and time-dependent area under the receiver operating characteristic curve (AUC). In addition, the calibration curve was used to evaluate the calibration of the model, and decision curve analysis (DCA) was used to assess the clinical utility of the model. All internal validations were conducted using the Bootstrap method, with a total of 1,000 resamples (16, 17). This helped to balance the differences in sample distribution, thereby more accurately validating the generalization ability and robustness of the model.

## 3 Results

### 3.1 Patient characteristics

A total of 105 pediatric patients who met the inclusion criteria were enrolled in this study, comprising 56 males and 49 females (Figure 1). The male-to-female incidence ratio was approximately 1.14:1. The median age at onset was 55 months (IQR: 29, 77). Among these patients, 16 (15.24%) presented with disease onset before 18 months of age, while 89 (84.76%) had an onset at or after 18 months of age. According to the INPC system, postoperative histopathological analysis revealed neuroblastoma (NB) in 29 cases, ganglioneuroblastoma nodular (GNBn) in 7 cases, ganglioneuroblastoma intermixed (GNBi) in 49 cases, and ganglioneuroma (GN) in 20 cases. Based on the INSS, 30 patients (28.57%) were classified as stage 1, 53 (50.48%) as stage 2, 12 (11.43%) as stage 3, and 10 (9.52%) as stage 4. No patient was identified as stage 4S in the study cohort. Baseline characteristics and relevant clinical data are summarized in Table 1.

**Table 1:** Demographic and clinical characteristics of pediatric patients with thoracic neuroblastoma.

Characteristics	Results [*n* = 105, n%/IQR (Q₁, Q₃)]
Age (months)	55 (29, 77)
Sex	
Male	56 (53.33)
Female	49 (46.67)
N-MYC	
No	81 (97.59)
Yes	2 (2.41)
Preoperative serum NSE (ng/mL)	19.85 (16.76, 27.83)
Preoperative serum LDH (U/L)	249.00 (223.00, 287.00)
Preoperative serum TP (g/L)	66.10 (63.20, 70.00)
Preoperative serum HDL (mmol/L)	1.29 (1.04, 1.54)
Preoperative serum Hb (g/L)	125.00 (118.00, 132.00)
MTD (cm)	5.10 (4.00, 7.00)
INPC	
GN	20 (19.05)
GNBi	49 (46.67)
GNBn	7 (6.67)
NB	29 (27.62)
INSS	
1	30 (28.57)
2	53 (50.48)
3	12 (11.43)
4	10 (9.52)

All the laboratory indicators were collected from the initial preoperative test results. NSE, neuron-specific enolase; LDH, lactate dehydrogenase; TP, total protein; HDL, high-density lipoprotein; Hb, hemoglobin concentration; MTD, maximum tumor diameter; INPC, International Neuroblastoma Pathology Classification system; INSS, International Neuroblastoma Staging System; IQR, interquartile range.

Among the 105 patients with thoracic neuroblastoma enrolled in the study, 64 (60.95%) exhibited a total of 93 IDRFs based on the final preoperative CT or MRI examination. Of these 64 patients, 44 had infiltrative IDRFs, 16 had vascular IDRFs, 9 had neurological IDRFs, 19 had extensive IDRFs, and 7 presented with preoperative pleural effusion. With regard to the distribution of IDRF counts, 42 patients (65.63%) had 1 IDRF, 16 patients (25.00%) had 2 IDRFs, 5 patients (7.81%) had 3 IDRFs, and 1 patient (1.56%) had 4 IDRFs. The proportion of IDRF counts is illustrated in Supplementary Figure 1.

### 3.2 Analysis of relevant factors influencing IDRFs

**Figure 2:**
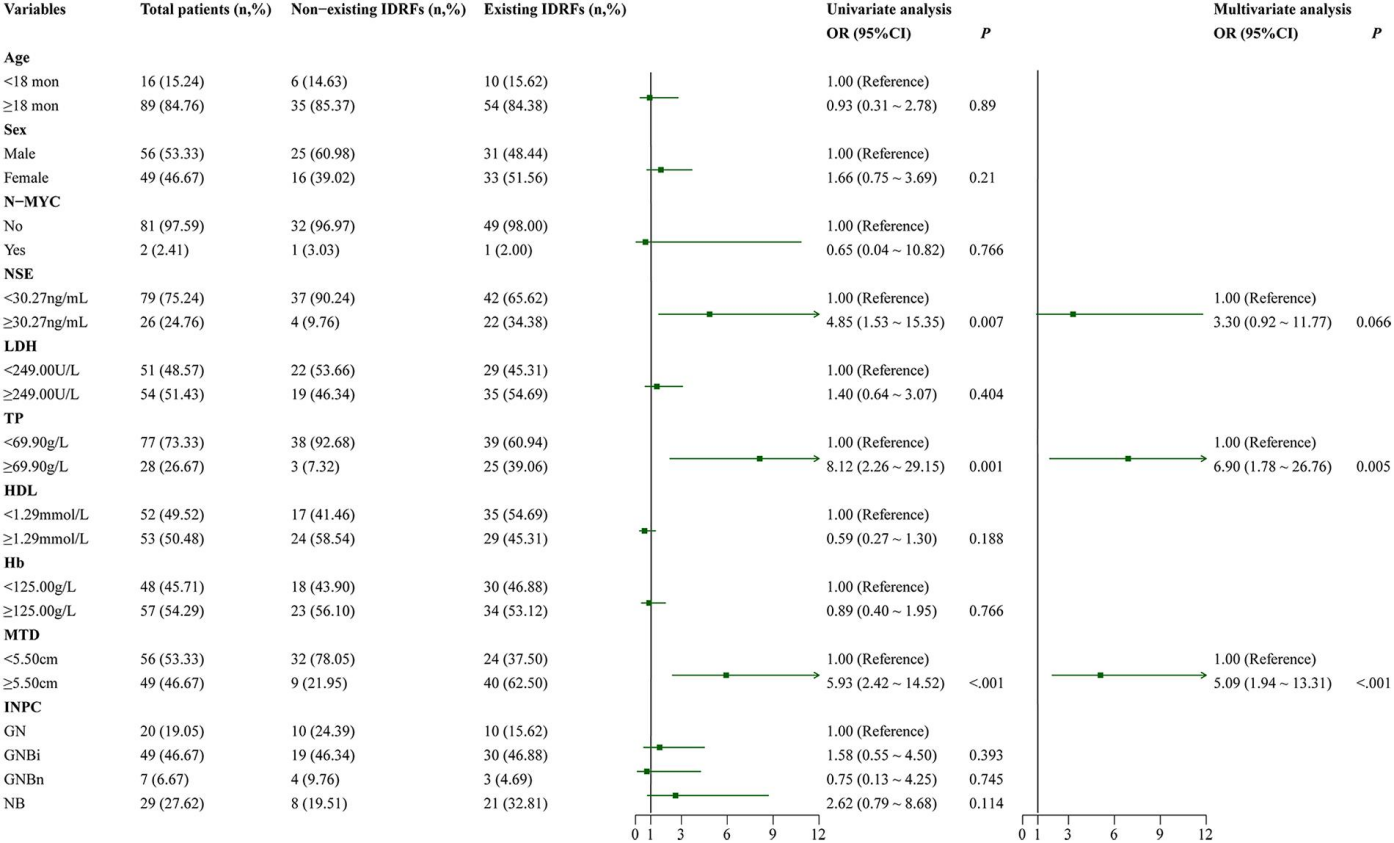
Forest plot of logistic regression analysis identifying factors associated with the existence of IDRFs. NSE: neuron-specific enolase; LDH: lactate dehydrogenase; TP: total protein; HDL: high-density lipoprotein; Hb: hemoglobin concentration; MTD: maximum tumor diameter; INPC: International Neuroblastoma Pathology Classification system.

To investigate potential factors influencing the occurrence of IDRFs, the logistic regression analysis was performed using demographic, clinical, and pathological characteristics of 105 pediatric patients with neuroblastoma as independent variables, with the presence or absence of IDRFs serving as the dependent variable. Before the analysis, the R package “Cutoff” was employed to determine the optimal cutoff values for NSE, TP, and MTD, which were identified as 30.27 ng/mL, 69.90 g/L, and 5.50 cm, respectively. LDH, HDL, and Hb levels were categorized based on their median values of 249.00 U/L, 1.29 mmol/L, and 125.00 g/L, respectively, while age was stratified at 18 months. Initially, univariate logistic regression analysis was conducted on the aforementioned variables. The results indicated that NSE ≥30.27 ng/mL (*p* = 0.007, OR = 4.85, 95% CI: 1.53–15.35), TP ≥ 69.90 g/L (*p* = 0.001, OR = 8.12, 95% CI: 2.26–29.15), and MTD ≥5.50 cm (*p* < 0.001, OR = 5.93, 95% CI: 2.42–14.52) were significantly associated with the occurrence of IDRFs. Variables with *p* < 0.10 were subsequently included in a multivariate logistic stepwise regression analysis, which identified TP level and MTD as independent risk factors for IDRFs. Patients with TP ≥ 69.90 g/L exhibited 6.90-fold increased risk of IDRFs compared to those with TP < 69.90 g/L, while patients with MTD ≥5.50 cm had 5.09-fold higher risk compared to those with a diameter <5.50 cm (Figure 2).

### 3.3 Correlation analysis between IDRFs and prognosis of thoracic neuroblastoma

#### 3.3.1 Survival characteristics

**Figure 3:**
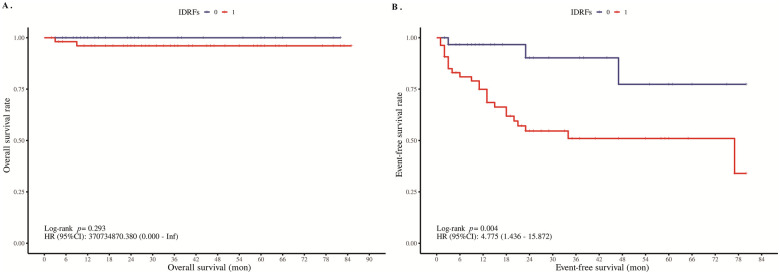
Kaplan–Meier curves of overall survival **(A)** and event-free survival **(B)** in thoracic neuroblastoma with or without IDRFs. “0” represents no; “1” represents yes.

Surgical therapy serves as the cornerstone in the management of localized neuroblastoma (10, 11). Thus, thoracoscopy was the preferred initial approach, enabling complete tumor resection and involved lymph node dissection. For paravertebral tumors with intraspinal invasion, large tumor volume, severe adhesion of the tumor to surrounding tissues and organs, or difficulty in tumor resection under thoracoscopy, thoracotomy was adopted. After excluding 20 patients who were postoperatively diagnosed with ganglioneuroma based on pathological examination, a total of 85 pediatric patients were enrolled for survival analysis. To evaluate the prognostic significance of IDRFs in neuroblastoma, the Kaplan–Meier method was employed to assess the association between the presence or absence of IDRFs and both overall survival (OS) and event-free survival (EFS). The follow-up period extended until December 2024. During the follow-up, 2 patients died (both confirmed as neuroblastoma by postoperative pathology), and 25 patients experienced tumor recurrence, metastasis, or the development of secondary malignancies (including 10 cases of GNBi, 2 cases of GNBn, and 13 cases of NB). According to the Kaplan–Meier survival analysis, the 5-year overall survival rate was 100% in the no-IDRFs group and 96.1% in the IDRFs group. However, no statistically significant difference in OS was observed between the two groups (*p* = 0.289, Figure 3A). For event-free survival, the 1-year EFS rate was 96.7%, the 3-year EFS rate was 90.2%, and the 5-year EFS rate was 77.3% in the no-IDRFs group, compared to 74.9%, 51.0%, and 51.0%, respectively, in the IDRFs group. A statistically significant difference in EFS was observed between the two groups (*p* < 0.05), as shown in Figure 3B.

#### 3.3.2 Analysis of factors associated with event-free survival (EFS)

Different types of IDRFs (infiltrative, vascular, neurological, and extensive) as well as varying numbers of IDRFs were incorporated into the analysis. The optimal cutoff values for NSE, LDH, TP, and MTD were determined using the R package “Cutoff”, yielding values of 34.21 ng/mL, 273.00 U/L, 70.00 g/L, and 4.80 cm, respectively. Age was categorized at 18 months, while HDL and Hb levels were stratified based on their median values of 1.28 mmol/L and 124.00 g/L, respectively. Using the presence or absence of adverse prognostic events as the grouping variable, the aforementioned indicators were compared and analyzed. Statistically significant differences between the two groups were observed in terms of EFS, INPC pathological classification, infiltrative IDRFs, vascular IDRFs, extensive IDRFs, number of IDRFs, NSE levels, LDH levels, TP levels, and MTD (*p* < 0.05), as summarized in Table 2.

**Table 2:** Analysis of the differences in variables between groups existing and non-existing adverse prognostic events.

Variables	Total (*n* = 85)	Non-existing adverse prognostic events (*n* = 58)	Existing adverse prognostic events (*n* = 27)	Statistic	*p*
Age, *n* (%)				*χ*^2^ = 1.31	0.253
<18 months	16 (18.82)	9 (15.52)	7 (25.93)		
≥18 months	69 (81.18)	49 (84.48)	20 (74.07)		
Sex, *n* (%)				*χ*^2^ = 0.11	0.742
Male	45 (52.94)	30 (51.72)	15 (55.56)		
Female	40 (47.06)	28 (48.28)	12 (44.44)		
NSE, *n* (%)				*χ*^2^ = 12.83	<.001
<34.21 ng/mL	67 (78.82)	52 (89.66)	15 (55.56)		
≥34.21 ng/mL	18 (21.18)	6 (10.34)	12 (44.44)		
LDH, *n* (%)				*χ*^2^ = 7.87	0.005
<273.00 U/L	53 (62.35)	42 (72.41)	11 (40.74)		
≥273.00 U/L	32 (37.65)	16 (27.59)	16 (59.26)		
TP, *n* (%)				*χ*^2^ = 7.11	0.008
<70.00 g/L	63 (74.12)	48 (82.76)	15 (55.56)		
≥70.00 g/L	22 (25.88)	10 (17.24)	12 (44.44)		
HDL, *n* (%)				*χ*^2^ = 2.91	0.088
<1.28 mmol/L	42 (49.41)	25 (43.10)	17 (62.96)		
≥1.28 mmol/L	43 (50.59)	33 (56.90)	10 (37.04)		
Hb, *n* (%)				*χ*^2^ = 0.00	0.991
<124.00 g/L	41 (48.24)	28 (48.28)	13 (48.15)		
≥124.00 g/L	44 (51.76)	30 (51.72)	14 (51.85)		
MTD, *n* (%)				*χ*^2^ = 8.09	0.004
<4.80 cm	38 (44.71)	32 (55.17)	6 (22.22)		
≥4.80 cm	47 (55.29)	26 (44.83)	21 (77.78)		
INPC, *n* (%)				-	0.012
GNBi	49 (57.65)	39 (67.24)	10 (37.04)		
GNBn	7 (8.24)	5 (8.62)	2 (7.41)		
NB	29 (34.12)	14 (24.14)	15 (55.56)		
Infiltrative IDRFs, *n* (%)				*χ*^2^ = 6.08	0.014
No	48 (56.47)	38 (65.52)	10 (37.04)		
Yes	37 (43.53)	20 (34.48)	17 (62.96)		
Vascular IDRFs, *n* (%)				*χ*^2^ = 14.45	<.001
No	71 (83.53)	55 (94.83)	16 (59.26)		
Yes	14 (16.47)	3 (5.17)	11 (40.74)		
Neurological IDRFs, *n* (%)				*χ*^2^ = 0.00	1.000
No	76 (89.41)	52 (89.66)	24 (88.89)		
Yes	9 (10.59)	6 (10.34)	3 (11.11)		
Extensive IDRFs, *n* (%)				*χ*^2^ = 10.64	0.001
No	68 (80.00)	52 (89.66)	16 (59.26)		
Yes	17 (20.00)	6 (10.34)	11 (40.74)		
Numbers of IDRFs, *n* (%)				—	<.001
0	31 (36.47)	28 (48.28)	3 (11.11)		
1	33 (38.82)	23 (39.66)	10 (37.04)		
2	15 (17.65)	7 (12.07)	8 (29.63)		
≥3	6 (7.06)	0 (0.00)	6 (22.22)		

NS, neuron-specific enolase; LDH, lactate dehydrogenase; TP, total protein; HDL, high-density lipoprotein; Hb, hemoglobin concentration; MTD, maximum tumor diameter; INPC, International Neuroblastoma Pathology Classification system; *χ*^2^, Chi-square test; “—”, Fisher exact test.

Subsequently, both univariate and multivariate Cox regression analyses were performed to evaluate the predictive value of these factors on event-free survival (EFS) in patients with neuroblastoma. Univariate Cox regression analysis revealed that INPC pathology classified as neuroblastoma (*p* = 0.010, HR = 2.87, 95% CI: 1.28–6.42), presence of infiltrative IDRFs (*p* = 0.038, HR = 2.29, 95% CI: 1.05–5.00), vascular IDRFs (*p* < 0.001, HR = 5.60, 95% CI: 2.53–12.40), extensive IDRFs (*p* = 0.006, HR = 2.94, 95% CI: 1.36–6.36), presence of two IDRFs (*p* = 0.008, HR = 6.12, 95% CI: 1.62–23.12), presence of three or more IDRFs (*p* < 0.001, HR = 17.37, 95% CI: 4.24–71.20), NSE ≥ 34.21 ng/mL (*p* < 0.001, HR = 4.54, 95% CI: 2.07–9.95), LDH ≥ 273.00 U/L (*p* = 0.003, HR = 3.27, 95% CI: 1.50–7.10), TP ≥ 70.00 g/L (*p* = 0.009, HR = 2.81, 95% CI: 1.30–6.10), and MTD ≥ 4.80 cm (*p* = 0.009, HR = 3.33, 95% CI: 1.34–8.27) were significantly associated with EFS in patients with neuroblastoma. All variables with *p* < 0.10 in the univariate analysis were included in the multivariate Cox regression. The optimal model variables were selected using bidirectional stepwise regression based on the AIC, identifying infiltrative IDRFs, vascular IDRFs, LDH ≥273.00 U/L, and TP ≥ 70.00 g/L as independent prognostic factors influencing EFS (Table 3). The multicollinearity analysis was conducted for these four variables, and their variance inflation factor (VIF) values were calculated as 1.885, 1.745, 1.235, and 1.175, respectively, all below 5, indicating no significant multicollinearity among the selected factors.

**Table 3:** Cox regression analysis of factors related to EFS in pediatric patients with thoracic neuroblastoma.

	Univariate analysis			Multivariate analysis		
Variables	*β*	HR (95%CI)	*p*	*β*	HR (95%CI)	*p*
Age, *n* (%)						
<18 months		1.00 (Reference)				
≥18 months	−0.27	0.76 (0.32–1.82)	0.538			
Sex, *n* (%)						
Male		1.00 (Reference)				
Female	0.07	1.07 (0.49–2.32)	0.861			
NSE, *n* (%)						
<34.21 ng/mL		1.00 (Reference)				
≥34.21 ng/mL	1.51	4.54 (2.07–9.95)	<.001			
LDH, *n* (%)						
<273.00 U/L		1.00 (Reference)			1.00 (Reference)	
≥273.00 U/L	1.18	3.27 (1.50–7.10)	0.003	0.90	2.47 (1.09–5.58)	0.030
TP, *n* (%)						
<70.00 g/L		1.00 (Reference)			1.00 (Reference)	
≥70.00 g/L	1.03	2.81 (1.30–6.10)	0.009	0.96	2.61 (1.19–5.75)	0.017
HDL, *n* (%)						
<1.28 mmol/L		1.00 (Reference)				
≥1.28 mmol/L	−0.65	0.52 (0.24–1.15)	0.105			
Hb, *n* (%)						
<124.00 g/L		1.00 (Reference)				
≥124.00 g/L	−0.02	0.98 (0.46–2.10)	0.969			
MTD, *n* (%)						
<4.80 cm		1.00 (Reference)				
≥4.80 cm	1.20	3.33 (1.34–8.27)	0.009			
INPC, *n* (%)						
GNBi		1.00 (Reference)				
GNBn	0.21	1.23 (0.27–5.68)	0.787			
NB	1.05	2.87 (1.28–6.42)	0.010			
Infiltrative IDRFs, *n* (%)						
No		1.00 (Reference)			1.00 (Reference)	
Yes	0.83	2.29 (1.05–5.00)	0.038	0.63	1.87 (0.85–4.14)	0.122
Vascular IDRFs, *n* (%)						
No		1.00 (Reference)			1.00 (Reference)	
Yes	1.72	5.60 (2.53–12.40)	<.001	1.35	3.86 (1.68–8.90)	0.002
Neurological IDRFs, *n* (%)						
No		1.00 (Reference)				
Yes	0.20	1.22 (0.37–4.07)	0.747			
Extensive IDRFs, *n* (%)						
No		1.00 (Reference)				
Yes	1.08	2.94 (1.36–6.36)	0.006			
Numbers of IDRFs, *n* (%)						
0		1.00 (Reference)				
1	1.09	2.99 (0.82–10.86)	0.097			
2	1.81	6.12 (1.62–23.12)	0.008			
≥3	2.86	17.37 (4.24–71.20)	<.001			

NSE, neuron-specific enolase; LDH, lactate dehydrogenase; TP, total protein; HDL, high-density lipoprotein; Hb, hemoglobin concentration; MTD, maximum tumor diameter; INPC, International Neuroblastoma Pathology Classification system; HR, Hazard ratio; 95% CI, 95% confidence interval; *β*, regression coefficient.

#### 3.3.3 Development of nomogram for predicting EFS

**Figure 4:**
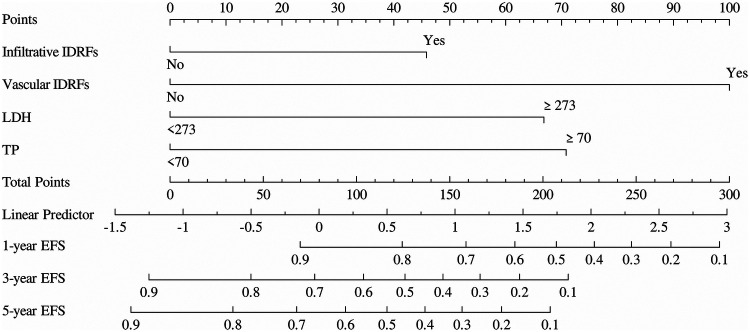
Nomogram for predicting EFS in pediatric patients with thoracic neuroblastoma. LDH: lactate dehydrogenase; TP: total protein.

The predictive nomogram model for EFS was developed based on four independent prognostic factors identified through univariate and multivariate Cox regression analyses. In this model, the presence of infiltrative IDRFs was assigned a score of 46.67, while their absence received a score of 0; the presence of vascular IDRFs was assigned 100 points, and absence received 0. For LDH levels ≥273.00 U/L, a score of 66.67 was assigned, whereas levels below this threshold received 0. Similarly, TP levels ≥70.00 g/L were assigned 71.11 points, and lower levels received 0. Using the nomogram, the total score from one or more prognostic factors can be calculated to estimate the 1-year, 3-year, and 5-year EFS rates in thoracic neuroblastoma. The lower total score indicates a reduced risk of adverse prognostic events, whereas a higher score corresponds to an increased risk (Figure 4).

#### 3.3.4 Validation and performance of the nomogram

**Figure 5:**
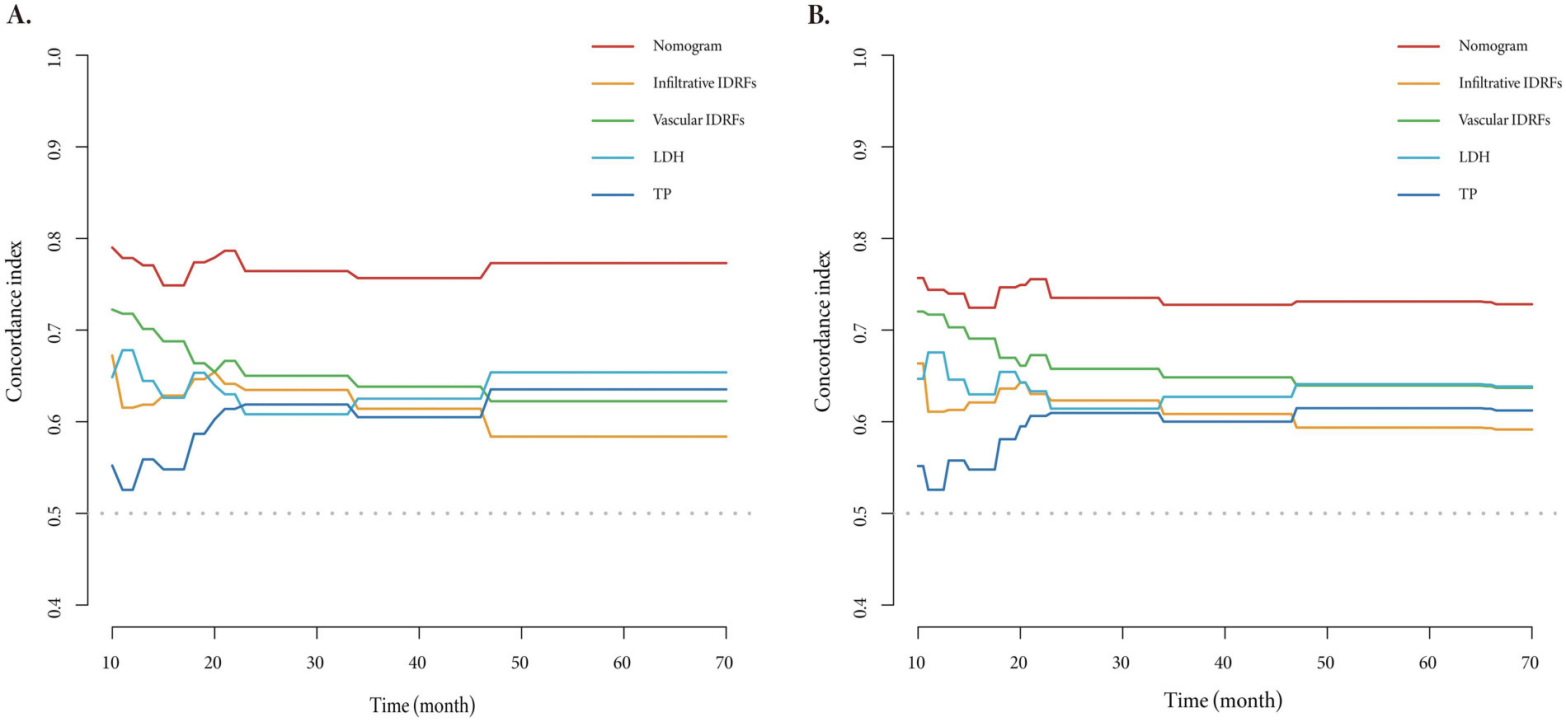
The time-dependent AUC curves of the nomogram for EFS **(A)** and the results of internal validation using the bootstrap method **(B)** compared with infiltrative IDRFs, vascular IDRFs, LDH ≥273.00 U/L, and TP ≥ 70.00 g/L. LDH, lactate dehydrogenase; TP, total protein.

To evaluate the discriminatory ability of the model, we calculated the concordance index (C-index) and time-dependent AUC curves. The results indicated that the overall C-index of the prognostic model was 0.77 (95% CI: 0.69–0.86), which was higher than 0.70. Additionally, the time-dependent AUC curves demonstrated that, compared with any single prognostic marker, the model exhibited superior prognostic accuracy in predicting 1-year, 3-year, and 5-year EFS rates in tumor patients (Figure 5A). Similar results were also observed when internal validation was conducted by resampling 1,000 times using the Bootstrap method (Figure 5B).

**Figure 6:**
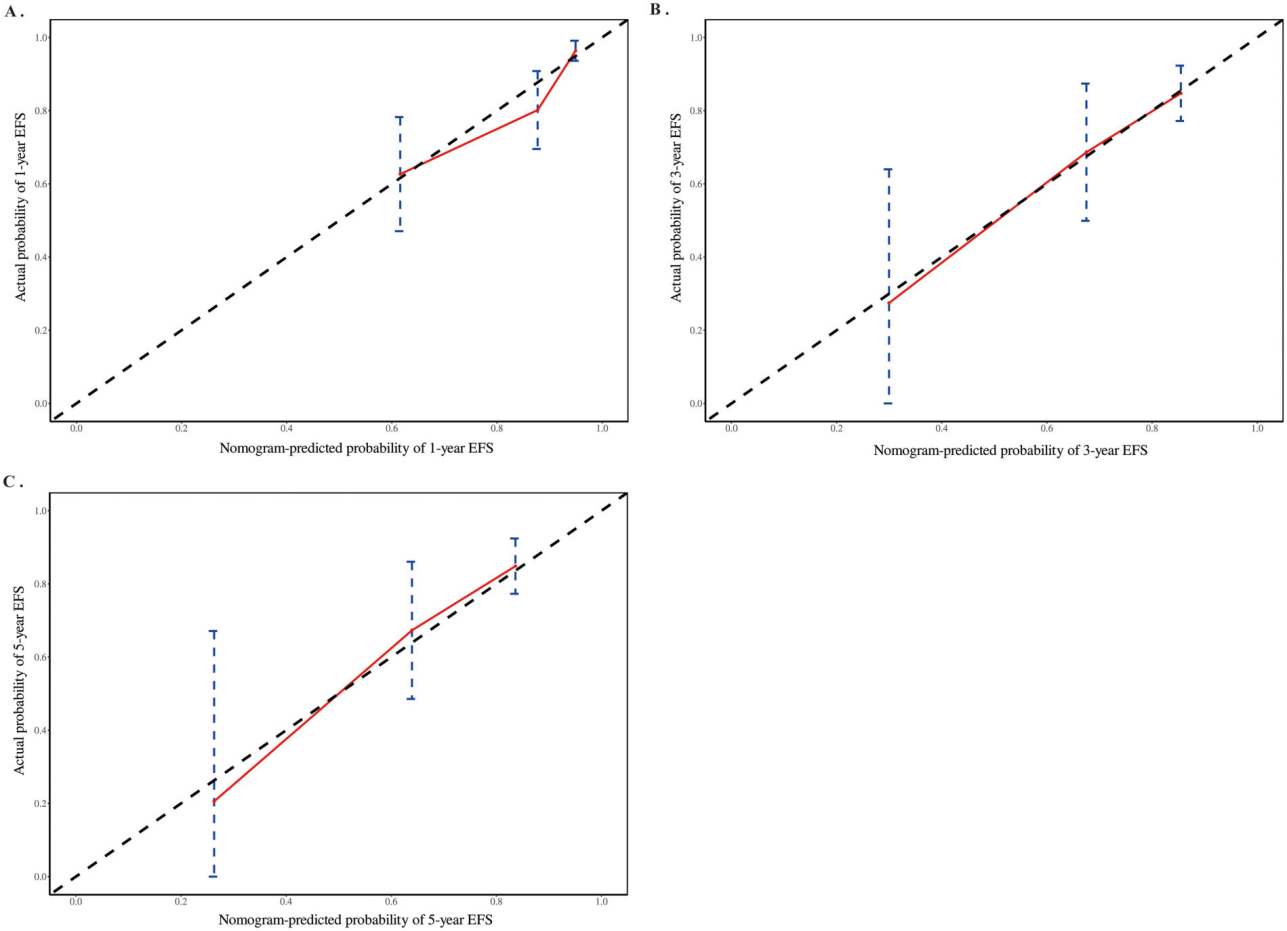
Calibration curves evaluating the calibration of nomogram for 1-year **(A)**, 3-year **(B)**, and 5-year **(C)** EFS.

Calibration curves were constructed to assess the calibration of the model in predicting 1-year, 3-year, and 5-year EFS rates. The results revealed a high degree of overlap between the predicted survival curves (red solid lines) and the ideal reference curves (black dashed lines) for 1-year, 3-year, and 5-year EFS, indicating a strong agreement between the model-predicted and observed survival probabilities (Figure 6).

**Figure 7:**
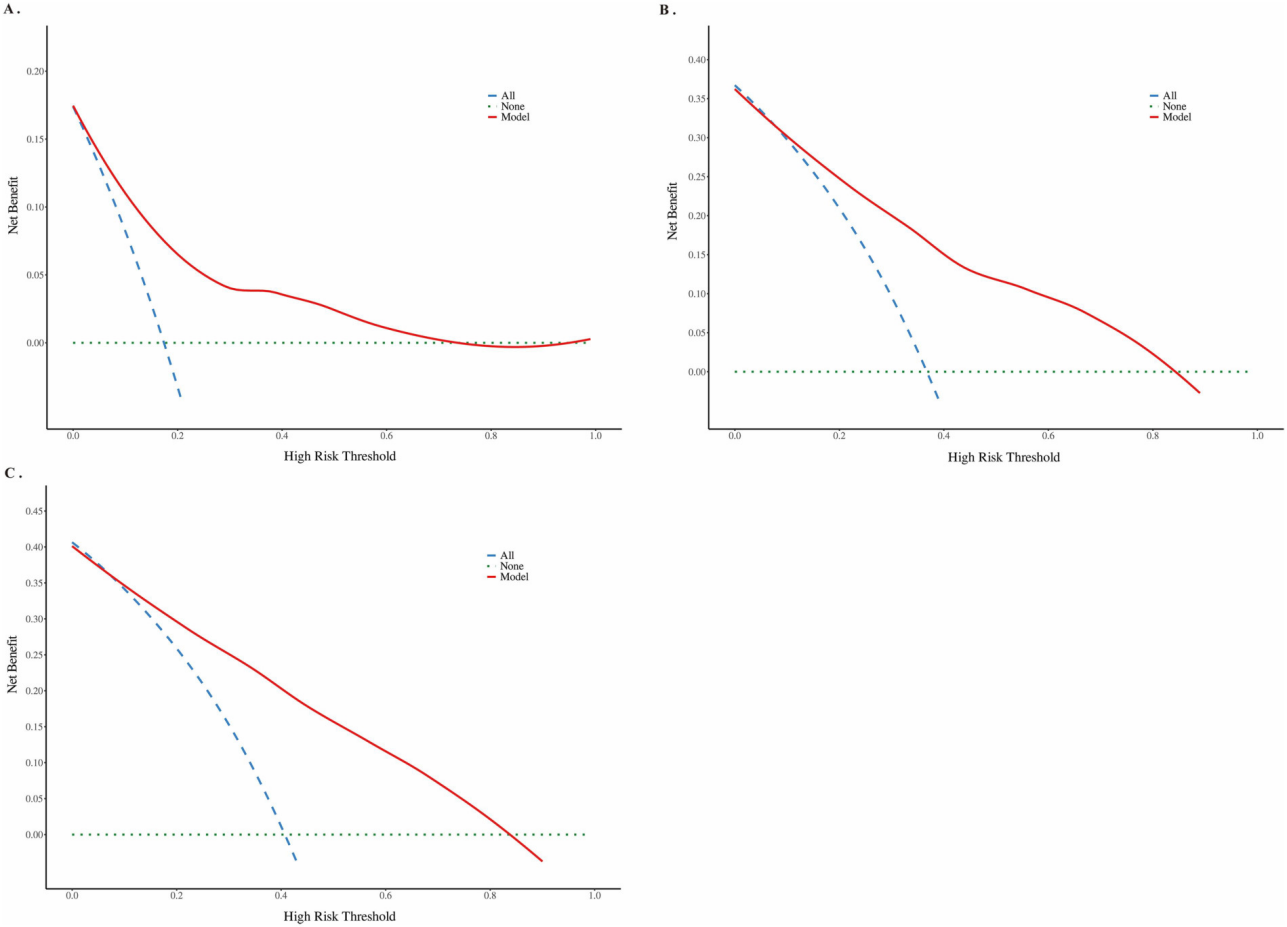
DCA curves evaluating the clinical utility of nomogram for 1-year **(A)**, 3-year **(B)**, and 5-year **(C)** EFS.

The clinical utility of the prediction model was further evaluated using DCA curves. In DCA curves, the *x*-axis represents the threshold probability, and the *y*-axis represents the net benefit. The green horizontal line and the blue diagonal line represent two extreme scenarios: the former assumes that no patients have risk factors, while the latter assumes that all patients have all risk factors, thereby ignoring individual variability (Figure 7). The results showed that the model achieved a favorable net benefit when the threshold probabilities for predicting 1-year, 3-year, and 5-year EFS were within the ranges of approximately 0.02–0.73, 0.08–0.84, and 0.09–0.85, respectively. These findings indicate that the model has potential clinical applicability.

## 4 Discussion

Thoracic neuroblastoma predominantly arises from the sympathetic ganglion regions within the posterior mediastinum, particularly the paravertebral groove, with an estimated incidence of 15%–20% (18, 19). Previous studies have demonstrated that neuroblastomas originating from different anatomical sites display marked heterogeneity in clinical features, biological behavior, and prognostic outcomes. Therefore, the present study retrospectively collected clinical and prognostic data from a single-center cohort of pediatric patients with thoracic neuroblastoma, with a specific focus on the distribution of IDRFs and their prognostic significance. The median age at diagnosis in this cohort was 55 months, with 89 patients (84.76%) diagnosed at or above 18 months of age. In comparison, previous studies have reported a median diagnostic age of approximately 2 years old (20), with nearly 40% of neuroblastoma cases diagnosed before the age of 1 year (18, 21). Furthermore, approximately 50% of thoracic neuroblastoma cases typically present at ≥18 months of age (9, 22). These comparisons suggested that the current cohort represented a relatively older diagnostic age group. Regarding histopathological classification, GNBi was the most frequently observed subtype, accounting for 46.67%, followed by classical NB at 19.05%. The observed findings align with the pathological distribution patterns reported by Yang S et al. (23), yet they contrasted with the results presented by Demir et al. (24) and Butzer et al. (9), which may indicate potential variations in the biological behavior of thoracic neuroblastoma across different populations and geographic regions. In contrast, the distribution of INSS staging and MYCN gene amplification in this study was consistent with previously published data.

Imaging-defined risk factors (IDRFs) evolved from the surgical risk factors (SRFs) previously established by the International Society of Pediatric Oncology-European Neuroblastoma Group (SIOPEN) to evaluate the feasibility and safety of surgical resection (25). Given their critical role in predicting surgical outcomes and clinical prognosis, IDRFs have garnered increasing attention in recent research. In this study, the classification system proposed by Delforge et al. (13) was applied to categorize thoracic neuroblastoma-related IDRFs into four major categories: infiltrative, vascular, neurological, and extensive. Among the 105 patients in the study cohort, 64 (60.95%) were identified as having at least one IDRF based on the final preoperative imaging evaluation. Regarding the number of IDRFs, the majority of patients exhibited a single IDRF (42 cases, 65.63%). In terms of type distribution, infiltrative IDRFs were the most frequently observed (44 cases, 68.75%), followed by vascular IDRFs (19 cases, 29.69%). To further investigate potential factors associated with the presence of IDRFs, demographic characteristics, pathological findings, preoperative laboratory tests, and imaging results were analyzed. Univariate and multivariate logistic regression analyses identified serum TP ≥ 69.90 g/L and MTD ≥ 5.50 cm as independent predictors of IDRF presence. Previous studies examining factors associated with IDRFs (26–28) have consistently highlighted tumor size as a key influencing factor, a finding corroborated in this study. Specifically, patients with tumors size ≥5.50 cm exhibited 5.09-fold increased likelihood of harboring IDRFs compared to those with smaller tumors. However, variations in patient age, sample size, clinical staging, and imaging modalities across studies led to discrepancies in the proposed tumor size thresholds. Therefore, future multicenter studies with larger cohorts are warranted to further validate these findings.

Another critical factor influencing the presence of IDRFs was the level of serum TP in patients. As a key biochemical marker, TP reflects various physiological and pathological conditions, including nutritional status, immune function, and inflammatory responses (29–31). It plays a significant role in evaluating disease severity, hepatic and renal function, and clinical prognosis. Moreover, due to its well-established testing methodology, cost-effectiveness, and wide clinical applicability, TP measurement is routinely performed in clinical settings. Previous studies have indicated that during carcinogenesis, elevated oxidative stress levels combined with impaired antioxidant defenses may lead to alterations in serum protein concentrations. More et al. (32) reported that increased serum protein levels may be associated with inflammatory responses in oral malignant tumors. Specifically, globulin, a major component of TP, was found to be significantly elevated in patients with oral cancer, suggesting that this increase may be attributed to its role as an acute-phase reactant. Similarly, Nimbal et al. (33) observed significantly higher TP levels in patients with smokeless tobacco-induced oral malignancies compared to those with precancerous lesions. Ghalehsari et al. (34) also reported elevated TP levels in patients with multiple myeloma, further supporting the association between TP and malignancy. Recent studies demonstrated that the neuroblastoma microenvironment contains a substantial number of immune and stromal cells, including M2 macrophages, natural killer T cells (NKT), tumor-infiltrating T lymphocytes (TIL-T), and cancer-associated fibroblasts (CAF) (35–37). These cells secreted various cytokines and proteases that suppress anti-tumor immunity, promote local inflammation, and facilitate tumor progression and metastasis. These findings suggested that TP levels might serve as a potential biomarker for neuroblastoma, reflecting disease severity. In our study, patients with TP ≥ 69.90 g/L exhibited 6.90-fold increased risk of harboring IDRFs compared to those with TP < 69.90 g/L. This association might be attributed to TP's capacity to reflect tumor burden and the degree of malignancy. Higher TP levels were indicative of more aggressive tumor biology, which in turn increases the probability of IDRFs occurrence.

The prognosis of patients with neuroblastoma has long been a central focus for clinical researchers. With the development and clinical application of novel therapeutic strategies, including immunotherapy, stem cell transplantation, and CAR-T cell therapy (38–40), the 5-year OS for patients with neuroblastoma has shown improvement over the past three decades (41). A recent population-based study reported that the 5-year survival rate for pediatric patients with neuroblastoma in the United States reached 79.7% by 2019 (42). Nevertheless, existing literature suggested that even among postoperative survivors, long-term outcomes and quality of life are not optimistic (43). In the present study, the 5-year OS was 100% in the group without imaging-defined risk factors (IDRFs) and 96.1% in the group with IDRFs. These results were consistent with previously published findings (44–46), yet higher than the survival rates reported for patients with high-risk neuroblastoma by Espinoza et al. (47). This suggested that thoracic neuroblastomas might generally carry a more favorable prognosis. However, a statistically significant difference in EFS was observed between the two groups (*p* < 0.05). Through group difference analysis and multivariate Cox regression analysis based on the Akaike information criterion (AIC), infiltrative IDRFs, vascular IDRFs, LDH ≥273.00 U/L, and TP ≥ 70.00 g/L were identified as independent prognostic factors influencing EFS in pediatric patients.

Numerous studies have demonstrated the significant prognostic value of imaging-defined risk factors (IDRFs) in neuroblastoma. A meta-analysis conducted by Parhar et al. revealed that patients with positive IDRFs exhibited significantly lower five-year OS and EFS compared to those without IDRFs (48). This finding was further supported by the most recent study from Yang et al. (28). Moreover, emerging evidence suggested that the presence of IDRFs may be associated with specific clinical and biological features of neuroblastoma. Factors such as larger tumor volume, MYCN gene amplification, and elevated serum levels of NSE and LDH have been implicated in this association (27, 28, 41). Notably, recent investigations indicated that not all IDRF subtypes demonstrated equivalent prognostic significance. Features such as major vascular encasement and intraspinal tumor infiltration were often indicative of highly aggressive tumor behavior and were associated with a poorer prognosis (13, 49).

For focal neuroblastoma, surgery is the cornerstone of treatment (10, 11). There have been numerous previous studies demonstrated that the presence of IDRFs was consistently associated with increased surgical complexity, prolonged postoperative hospitalization, reduced rates of gross-total resection, and higher incidence of perioperative complications (47, 50–53). Especially for tumors with vascular IDRFs, the tumor volume was often larger, and the difficulty of separating the tumor from the vessels was higher, which undoubtedly brought extremely high difficulty and risk to thoracoscopic surgery and increases the rate of incomplete tumor resection (44, 53). Saksiri et al. reported that there was a significant difference in the occurrence of surgical complications between the groups with or without IDRFs, with approximately over 60% of neuroblastoma patients with IDRFs experiencing surgery-related complications (52). These factors undoubtedly worsen the prognosis of the patients and have a significant impact on the treatment outcome.

In this study, we first integrated IDRFs into the development of a nomogram designed to predict EFS in patients with thoracic neuroblastoma. The calibration curves for 1-year, 3-year, and 5-year EFS demonstrated excellent agreement with the ideal reference curves, indicating good calibration and prediction accuracy. Furthermore, the overall C-index of the prognostic model exceeded 0.70, and DCA curves revealed that the model provided a favorable net benefit across a broad range of threshold probabilities. Therefore, this nomogram might serve as a valuable tool for estimating EFS in patients with thoracic neuroblastoma, assisting clinicians in developing more individualized and precise treatment strategies, thereby potentially improving long-term survival and quality of life in affected children.

Certainly, this study also has some limitations. First, the low prevalence of MYCN amplification in this cohort precluded its inclusion in the prognostic analysis. Moreover, due to economic cost and diagnostic technology limitations, other established prognostic biomarkers, such as segmental chromosomal aberrations (SCAs) and anaplastic lymphoma kinase (ALK) status, were not evaluated, resulting in the absence of relevant data. This has limited the reliability and generalizability of the prognostic analysis in this study. Second, the sample size of this study was limited and all data are from a single center, which might lead to potential selection bias. Therefore, future multi-center studies with larger sample sizes are warranted to validate our findings. Finally, the current prediction model was specifically developed for thoracic neuroblastoma and might not be directly applicable to tumors originating from other anatomical sites. So, further investigations are warranted to assess its performance in anatomical locations.

## 5 Conclusions

Thoracic neuroblastoma exhibited distinct demographic, clinical, and pathological characteristics compared to nonthoracic tumors. TP level and maximum tumor diameter were independently associated with the presence of IDRFs. IDRFs significantly fluenced EFS in pediatric patients with thoracic neuroblastoma, with infiltrative IDRFs, vascular IDRFs, elevated LDH, and increased TP levels identified as independent prognostic risk factors for EFS. The nomogram model developed based on these risk factors demonstrated robust predictive performance and held potential for clinical application.

## Data Availability

The raw data supporting the conclusions of this article will be made available by the authors, without undue reservation.
