# Supplementary figures and images for: Association of image-defined risk factors with clinical features in thoracic neuroblastoma and the development of a prognostic prediction model
Source: Front Pediatr. 2026 Apr 22;14:1793866. doi: 10.3389/fped.2026.1793866 (PMC13144076; doi:10.3389/fped.2026.1793866)

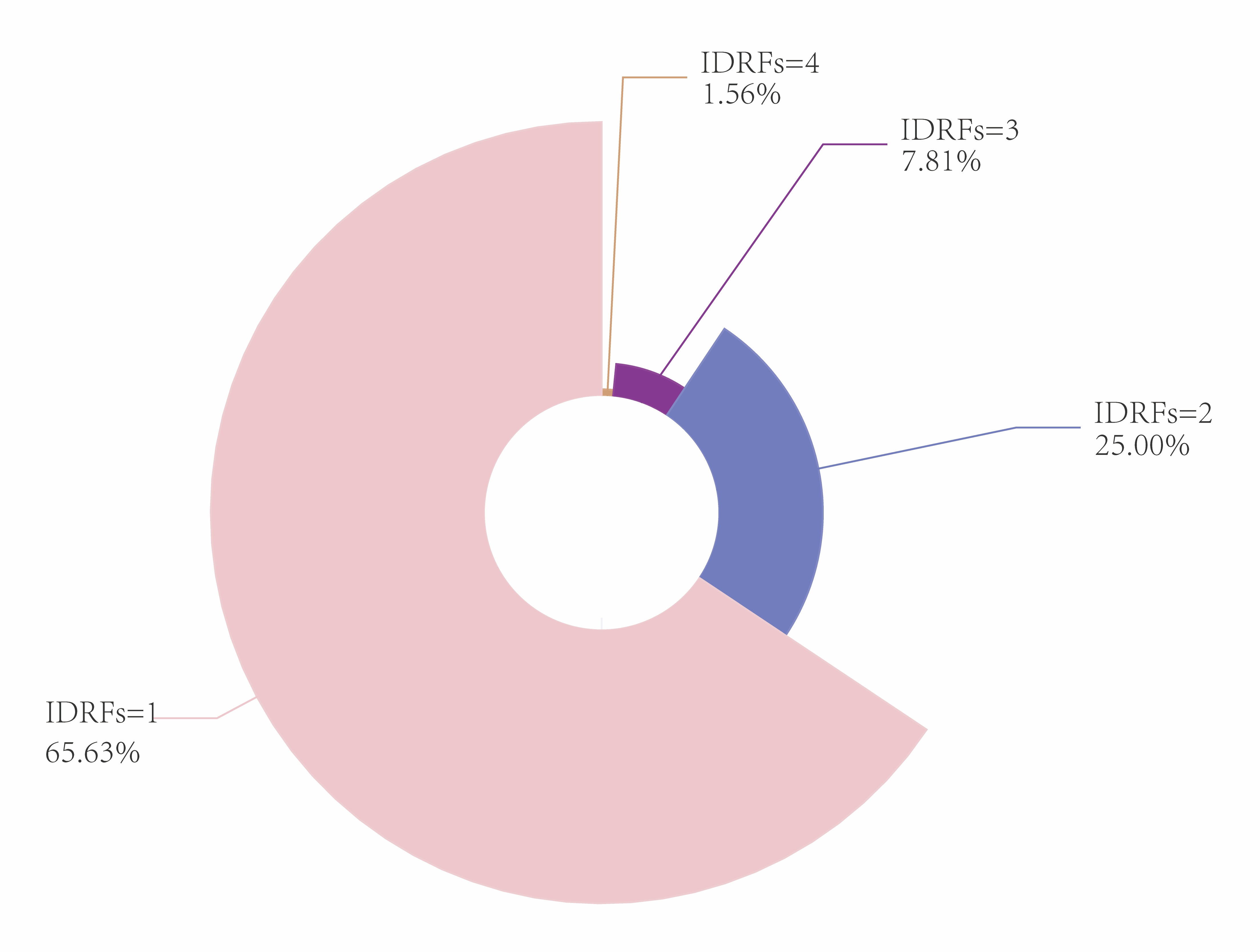

Supplement: Supplementary file 1 [file Image1.tif]
